# Development and Evaluation of a Multiplex Quantitative Real-Time Polymerase Chain Reaction for Hookworm Species in Human Stool

**DOI:** 10.4269/ajtmh.18-0276

**Published:** 2018-09-17

**Authors:** Sze Fui Hii, Dammika Senevirathna, Stacey Llewellyn, Tawin Inpankaew, Peter Odermatt, Virak Khieu, Sinoun Muth, James McCarthy, Rebecca J. Traub

**Affiliations:** 1 Faculty of Veterinary and Agricultural Sciences, University of Melbourne, Parkville, Australia;; 2 Clinical Tropical Medicine Laboratory, QIMR Berghofer Medical Research Institute, Herston, Australia;; 3 Department of Parasitology, Faculty of Veterinary Medicine, Kasetsart University, Bangkok, Thailand;; 4 Department of Epidemiology and Public Health, Swiss Tropical and Public Health Institute, Basel, Switzerland;; 5 University of Basel, Basel, Switzerland;; 6 National Centre for Parasitology, Entomology, and Malaria Control, Phnom Penh, Cambodia

## Abstract

Hookworm disease caused by *Necator americanus*, *Ancylostoma duodenale*, and *Ancylostoma ceylanicum* affects half a billion people worldwide. The prevalence and intensity of infection of individual hookworm species are vital for assessing morbidity and generating targeted intervention programs for their control. The present study aims to evaluate a multiplex real-time quantitative PCR (qPCR) assay to determine the prevalence and egg intensity of all three hookworm species and compare this with standard microscopy and published genus-based conventional and real-time multiplex qPCRs. Performance of the diagnostic assays was evaluated using DNA extracted from 192 fecal samples collected as part of a soil-transmitted helminth (STH) survey in northern Cambodia. The prevalence of hookworms as detected by the multiplex hookworm qPCR of 84/192 (43.8%) was significantly higher than that using microscopy of 49/192 (25.5%). The hookworm multiplex qPCR showed very good agreement for the detection of both *N. americanus* (Kappa 0.943) and *Ancylostoma* spp. (Kappa 0.936) with a multiplex STH qPCR. A strong and moderate quantitative correlation between cycle threshold and eggs per gram (EPG) feces was obtained for the hookworm qPCR for seeded DNA egg extracts (*R*^2^ ≥ 0.9004) and naturally egg-infected individuals (*R*^2^ = 0.6848), respectively. The newly developed hookworm quantitative multiplex qPCR has the potential for application in anthelmintic efficacy trials and for monitoring the success of mass deworming programs targeting individual species of anthroponotic and zoonotic hookworms.

## INTRODUCTION

Hookworm disease, caused by blood-feeding worms residing in the small intestine, affects nearly half a billion people worldwide.^1^ Hookworms cause iron deficiency anemia and malnutrition, and in pregnant women, infection is associated with poor neonatal outcomes, including low birth weight and increased infant mortality. In children, infection may result in impaired physical and intellectual development.^2^

Three species of hookworms are capable of producing patent infections in humans: *Necator americanus*, *Ancylostoma duodenale*, and *Ancylostoma ceylanicum*. All three species have similar life cycles but have important differences in pathogenicity and host specificity. These differences may have important public health implications, such as assessing the success of intervention programs in a community. For example, *A. ceylanicum*, now recognized as the second most common hookworm species in the Asia Pacific region, is a zoonotic agent, with dogs and cats acting as primary reservoirs for human infection.^3,4^ Control of this hookworm necessitates a One Health approach. The presence of *A. duodenale* in a population causes higher morbidity through increased blood loss^5,6^ and results in significantly different seasonal and age-related epidemiology to the other species, owing to the ability of larvae to undergo tissue hypobiosis.^7,8^ Moreover, hookworm species may vary in their response to chemotherapeutic intervention^9^; however, current tools do not allow us to accurately assess this as part of chemotherapeutic efficacy trials.

Standard microscopy-based detection of hookworm eggs in fecal samples fail to differentiate the species of hookworms infecting humans, and are recognized to be insensitive compared with both conventional^4^ and real-time PCR.^10–12^ Thus, microscopic techniques may be inadequate for use in clinical trials or in monitoring and evaluating programs when infection intensity is low. Real-time multiplex and multi-parallel PCR assays for the differentiation of *Ancylostoma* spp. and *N. americanus* have advantages over parasitologic methods of being able to be batch processed in a high-throughput fashion and provide estimates of infection intensity by correlating average DNA concentration and egg counts.^11–14^ To date, however, real-time PCR assays have not been able to differentiate and quantitate all three species of human hookworms simultaneously without having to revert to conventional PCR followed by DNA sequencing^15^ or restriction fragment length polymorphism analysis of PCR products.^16^

The aim of the present study was to develop a multiplex quantitative PCR assay for the simultaneous detection of three species of hookworms that infect humans and to evaluate its diagnostic performance against standard microscopy and two published PCR assays for the detection of hookworm.^11,15^

## METHODS

### Multiplex PCR development.

The multiplex quantitative PCR assay for hookworm was designed to detect *N. americanus*, *A. ceylanicum*, *A. duodenale*/*Ancylostoma caninum*, and *Equine herpesvirus* type 4 (EHV4) as an internal control. The assay was performed using the previously published primers and probe for *N. americanus*^10^ and EHV4^17^ and newly designed primers and probes for *A. ceylanicum* and *A. duodenale*/*A. caninum* listed in Table 1.

**Table 1:** Primer and probe sets for the quantitative hookworm multiplex PCR

Target	Primer/Probe	Sequence 5′ to 3′	Size (bp)	Gene target	Conc. nM	Source
*Necator americanus*	NecF	CTGTTTGTCGAAC-GGTACTTGC	101	ITS2	250	10
	NecR	ATAACAGCGTGCA-CATGTTGC			250	
	NecP	Cy5-CTGTACTACG-CATTGTATAC-IBFQ			100	
*Ancylostoma* spp.	AncF	CGGGAAGGTTGG-GAGTATC	104	ITS1	300	This study
	AncR	CGAACTTCGCACA-GCAATC			300	This study
*Ancylostoma ceylanicum*	AncCeyP	FAM-CCGTTCCTGG-GTGGC-IBFQ			100	This study
*Ancylostoma duodenale*	AncDuoP	HEX-TCGTTACTG-GGTGACGG-IBFQ			100	This study
EHV	EHVFWD	GATGACACTAGCG-ACTTCGA	81	gB	40	15
	EHVREV	CAGGGCAGAAACC-ATAGACA			40	
	EHVProbe	ROX-TTTCGCGTGC-CTCCTCCAG-IBRQ			100	

EHV = *Equine herpesvirus*; ITS = internal transcribed spacer 2.

Nucleotide sequences for the internal transcribed spacer 1 gene for *A. ceylanicum* (GenBank accession nos. DQ780009 and DQ831517) and *A*. *duodenale* (GenBank accession no. EU344797) were obtained from GenBank and aligned using CLUSTALW (http://www.genome.jp/tools/clustalw/). A forward primer (AncF), a reverse primer (AncR), an *A. ceylanicum*–specific probe (AncCeyP), and an *A. duodenale*/*A. caninum*–specific probe (AncDuoP) (Table 1) were designed to amplify a target (104 bp) of both species. *Equine herpesvirus* type 4 was used as an amplification control. An assay was deemed a failure when the cycle threshold (Ct) value of the EHV PCR in the sample was greater by two or more cycles compared with the negative control EHV Ct value. Magnetic Induction Cycler (Bio Molecular Systems, Sydney, Australia) was used for the amplification, detection, and data analysis.

Minor modifications were made to previously published soil-transmitted helminth (STH) multiplex quantitative PCR (qPCR) protocols.^11^ The qPCR was conducted in 20 μL volumes containing GoTaq^®^ Probe qPCR Mastermix (Promega, Madison, WI), 1 μL of known quantity of EHV4, and 2 μL of template DNA. Quantitative PCR conditions included an initial denaturation step at 95°C for 2 minutes followed by 40 cycles of amplification using denaturation at 95°C for 15 seconds followed by extension at 60°C for 1 minute. The cycling threshold was held at 0.02 units for each multiplex PCR. The fluorescent threshold was set at 10% for *N. americanus*, *A. ceylanicum*, and *A. duodenale* and 20% for EHV4. All templates were run in duplicate.

Synthetic block gene fragments (IDT^®^ Technologies, Skokie, Illinois, USA) containing the DNA sequences targeted by the PCRs for *A. ceylanicum*, *A. duodenale*, and *N. americanus* were used as templates for optimization of the singleplex and multiplex assays and as positive controls. Nuclease-free water was used as negative control in all runs. Optimal concentration of primer pairs and probes were initially determined with standard curve analysis in singleplex PCR. Concentration of primer pairs and probes that demonstrated greatest sensitivity and highest efficiency was selected for progression into multiplex PCR format. The Ct of serial 10-fold dilution series of gBlock gene fragments of known DNA concentration of each worm target was compared in both separate and multiplex reactions.

### Assay quantification using seeded hookworm eggs.

Eggs of *N. americanus* (source, human; Queensland), *A. caninum* (source, dogs; Vietnam), and *A. ceylanicum* (source, dogs; Vietnam) were purified from 5 to 10 g stool using sucrose gradient centrifugation and suspended in two 50 μL aliquots of 1× phosphate-buffered saline and enumerated. Known quantities of eggs representing each hookworm species were subjected to 2-fold serial dilution ranging from 15 eggs to a maximum of 3,000 eggs (limited by egg counts in sourced fecal samples) and DNA extraction in duplicate using the Fecal Isolate II DNA extraction kit (Bioline, London, United Kingdom). Two microliters of DNA was added to each multiplex hookworm qPCR, also run in duplicate. Log_10_ transformations of absolute egg count for each hookworm species were plotted against Ct values to predict the ability of the assay to estimate absolute egg counts (in DNA extract) and original egg intensities in EPG feces for each hookworm species, assuming a 100% run efficiency. Absolute to original egg counts (EPG feces) were calculated by multiplying the absolute egg count by a factor of 4, as 250 mg of fecal sample was subjected to DNA extraction for the validation (field) study.

### Assay validation.

Genomic DNA isolated from faecal samples (n = 190) sourced from a previous cross-sectional study conducted in 2012^4^ in Cambodia and, that was previously used to validate a multiplex STH qPCR,^11^ were used to validate the current multiplex hookworm qPCR. DNA, was extracted from these samples in July 2013, and stored at −20°C for subsequent use. These fecal samples were previously screened using sodium nitrate flotation and the intensity of hookworms in EPG feces was determined.^4,18^ In addition, two *A. duodenale*–positive samples obtained from the same study site in Cambodia in July 2016 were added to the sample cohort, owing to the absence of *A. duodenale* DNA from the 2012 sample cohort. The diagnostic parameters of the multiplex hookworm qPCR assay were compared with 1) microscopy (sodium nitrate flotation); 2) multiplex conventional PCR, which detects *Necator* spp. and *Ancylostoma* spp. and differentiates genera based on amplicon size and *Ancylostoma* spp. based on DNA sequence analysis^15^; and 3) a real-time multiplex PCR for detection and quantification of *Necator* and *Ancylostoma* spp. as part of a multiplex STH real-time assay.^10,11^

The intensity of hookworm infections in EPG by sodium nitrate flotation was plotted against Ct values for the hookworm multiplex qPCR to assess the quantitative ability of the assay on field samples.

### Statistical analysis.

Analysis of results was performed using Excel 2008 (Microsoft Corp., Redmond, WA). Kappa statistics were calculated using GraphPad QuickCalcs (GraphPad Software, La Jolla, CA) to compare hookworm multiplex qPCR with those generated by microscopic examination and previous STH multiplex qPCR.

### Ethical considerations.

The field study in Cambodia^4^ was approved by the National Ethics Committee for Health Research, Ministry of Health, Cambodia (NECHR, #192), and Ethics Committee of the Cantons of Basel-Stadt and Baselland (EKBB, #18/12). Written informed consent was obtained from each participant before the start of the study. For participants between the ages of 2 and 18 years, written informed consent was obtained from the parents, legal guardian, or appropriate literate substitute. All participants were informed of the study’s purpose and procedures before enrolment. All parasitic infections diagnosed were treated as per the guidelines of the National Helminth Control Program of Cambodia.

## RESULTS

### Hookworm multiplex qPCR optimization.

**Figure 1.**
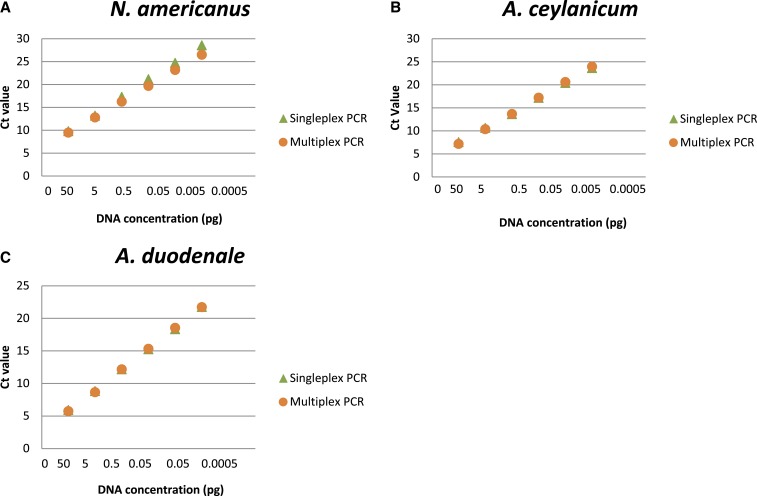
Singleplex and multiplex qPCR efficiencies. Assay optimization to compare the efficiencies of single vs. multiplex PCR assays using gBlock gene targets for (**A**) *Necator americanus*, (**B**) *Ancylostoma ceylanicum*, and (**C**) *Ancylostoma duodenale* fragments (IDT^®^ Technologies) as standard curve controls. Ct = cycle threshold. This figure appears in color at www.ajtmh.org.

The optimized primer and probe concentrations are listed in Table 1. The Ct values versus target DNA concentrations and reaction efficiencies and sensitivities obtained from serial dilutions of *N. americanus*, *A. ceylanicum*, and *A. duodenale* gBlock gene fragments (IDT^®^ Technologies, Skokie, IL) as singleplex and multiplex assays are shown in Figure 1. There were no observed differences in reaction efficiency or sensitivity (Figure 1) between singleplex and multiplex assays.

No cross-reaction in the qPCR assays was observed between hookworm species when singleplex and multiplex *N. americanus*, *A. ceylanicum*, and *A. duodenale* assays were assessed using gBlock gene fragments (IDT^®^ Technologies, Skokie, Illinois, USA) consisting of individual hookworm species targets, or with genomic DNA templates extracted from individual hookworm species (adult worms and egg-positive field samples). The specificity of singleplex and multiplex assays were also assessed using positive control genomic DNA from *Ascaris lumbricoides*, *Strongyloides stercoralis*, *Trichuris trichiura*, *Opisthorchis viverrini*, *Clonorchis sinensis*, *Haplorchis taichui*, *Hymenolepis nana*, *Taenia saginata*, *Taenia solium*, *Giardia duodenalis*, *Blastocystis hominis*, *Enterobius vermicularis*, *Cryptosporidium hominis*, and *Cryptosporidium parvum*. The PCR assays for *A. ceylanicum*, *A. duodenale*/*A. caninum*, *N. americanus*, and EHV4 amplified products exclusively from their corresponding DNA templates.

### Quantitative ability of hookworm multiplex qPCR assays.

**Figure 2.**
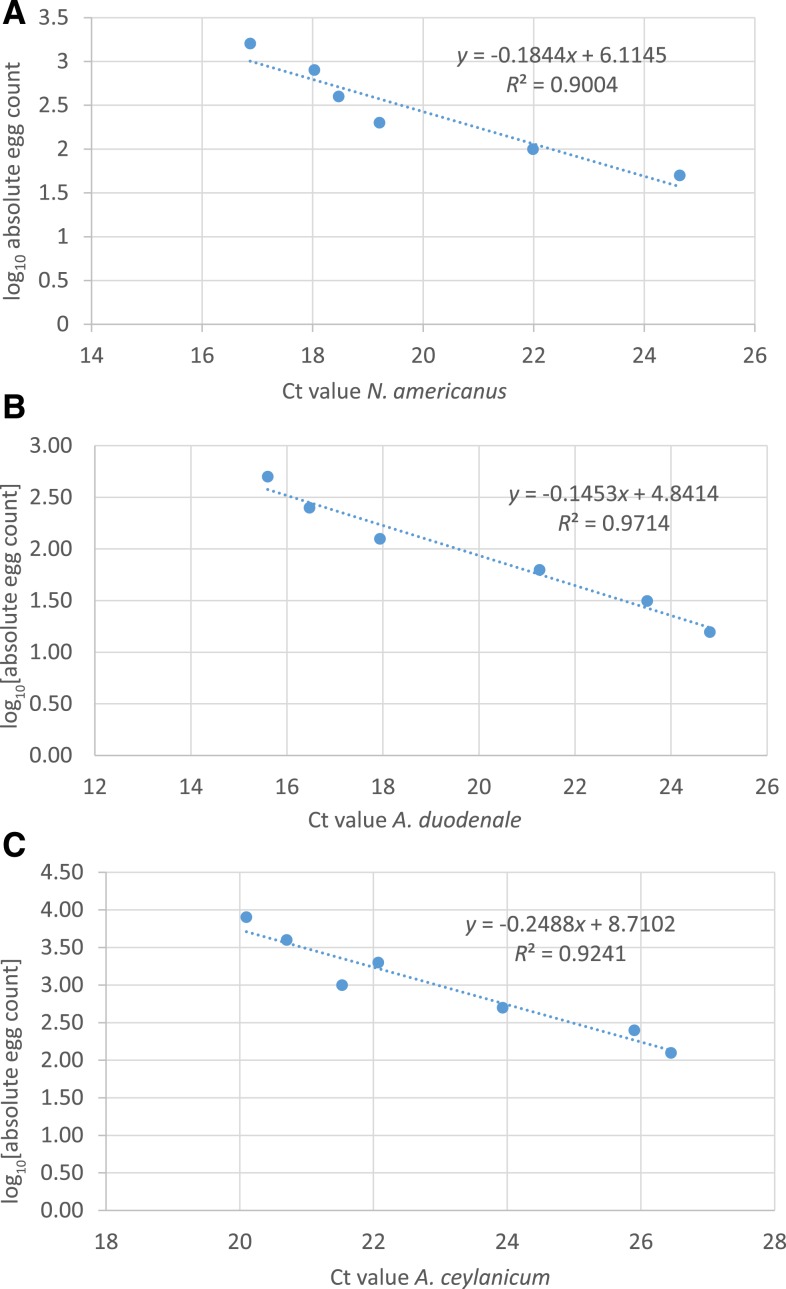
Cycle threshold (Ct) values plotted against known quantities of purified seeded eggs of (**A**) *Necator americanus*, (**B**) *Ancylostoma caninum*, and (**C**) *Ancylostoma ceylanicum*. An excellent correlation was displayed between seeded average egg counts and average Ct measured for *N. americanus*, *Ancylostoma duodenale/A. caninum*, and *A. ceylanicum* by the multiplex hookworm quantitative PCR. This figure appears in color at www.ajtmh.org.

The Ct values plotted against known seeded quantities of purified eggs of *N. americanus*, *A. caninum*, and *A. ceylanicum* eluted in 50 μL tris EDTA buffer and the conversion formulas for Ct to absolute egg counts (2 μL DNA/PCR assay) are shown in Figure 2A–C. There was a strong linear correlation between Ct values and the *N. americanus*, *A. caninum*, and *A. ceylancium* log_10_ absolute egg counts (*R*^2^ between 0.9004 and 0.9714).

### Performance of hookworm multiplex qPCR.

**Figure 3.**
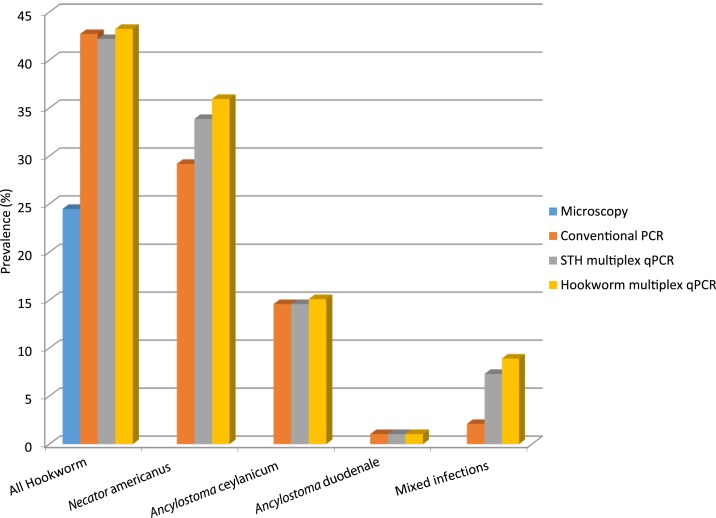
Hookworm prevalence by multiplex hookworm quantitative PCR (qPCR), multiplex soil-transmitted helminth (STH) qPCR^11^ conventional PCR,^4^ and sodium nitrate flotation (microscopy).^18^ Data presented combined for all 192 study participants, showing higher recorded percentage prevalence across all genera and species of hookworms, including mixed species infections, by multiplex hookworm qPCR.

Performance of the hookworm multiplex qPCR was evaluated using a total of 192 DNA templates extracted from previous hookworm surveys in Cambodia. Multiplex qPCR runs, positive controls, and EHV4 were detected and amplified at expected Ct values. Overall, the multiplex hookworm qPCR demonstrated higher detection rates for hookworms compared with microscopy- and genus-based conventional^15^ and real-time multiplex PCRs (Figure 3).^11^

**Figure 4.**
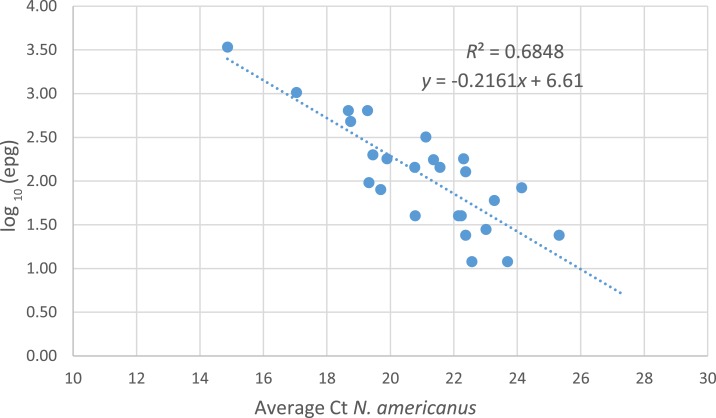
Relationship between cycle threshold (Ct) value for multiplex hookworm qPCR and fecal flotation derived egg counts (EPG) for *Necator americanus.* Microscopy negative Ct values not displayed. A moderate-to-good correlation was displayed between average *N. americanus* egg counts measured by sodium nitrate flotation and average Ct measured by the multiplex hookworm qPCR. EPG = eggs per gram. This figure appears in color at www.ajtmh.org.

### Comparison of the hookworm multiplex qPCR with sodium nitrate flotation.

Table 2 shows the detection of hookworm infection using microscopy and multiplex hookworm qPCR. The prevalence of hookworms as detected by the multiplex qPCR of 84/192 (43.2%) was significantly higher than those detected by microscopy of 48/192 (25.0%). Kappa statistics showed only moderate agreement between the two diagnostic techniques. Thirty-six or 18.75% of hookworm-positive samples detected by qPCR were missed by sodium nitrate flotation and microscopy. These comprised 24/69 (34.8%) of *N. americanus* infections and 12/29 (41.3%) of *A. ceylanicum* infections.

**Table 2:** Multiplex quantitative PCR and microscopy agreement statistics

	Multiplex PCR (this study)	Microscopy^4,18^		Total agreement (%)	Kappa* (agreement level)	Standard error of Kappa
		POS	NEG			
Hookworm	POS	48	36	156 (80.38)	0.590 (moderate)	0.056
	NEG	1	107			

NEG = negative; POS = positive.

* Kappa agreement level: *K* < 0.2, poor; 0.21–0.40, fair; 0.41–0.60, moderate; 0.61–0.80, good; 0.81–1.00, very good.

Kappa statistics (Table 3) show good agreement between the hookworm multiplex qPCR and the conventional PCR for the species of hookworms. The hookworm multiplex qPCR identified an additional 22 fecal samples positive for hookworm that were missed by conventional PCR. Eight samples (two for *N. americanus* and six for *A. ceylanicum*) were positive by conventional PCR but missed by the hookworm multiplex qPCR.

**Table 3:** Comparison of hookworm multiplex qPCR with multiplex STH qPCR and conventional PCR

	qPCR	STH qPCR		Total agreement (%)	Kappa* ± SE	cPCR		Total agreement (%)	Kappa* ± SE
		POS	NEG			POS	NEG		
*Necator americanus*	POS	64	5	184 (96.84)	0.931 ± 0.028	54	15	173 (91.05)	0.798 ± 0.046
	NEG	1	120			2	119		
*Ancylostoma ceylanicum*	POS	27	2	188 (98.43)	0.943 ± 0.035	21	7	177 (92.67)	0.707 ± 0.074
	NEG	1	161			7	156		
*Ancylostoma duodenale*	POS	2	0	2 (100%)	N/A	2	0	2 (100%)	N/A
	NEG	0	0			0	0		

N/A = Not applicable; NEG = negative; POS = positive; qPCR = quantitative PCR; STH = soil-transmitted helminth.

* Kappa agreement level: *K* < 0.2, poor; 0.21–0.40, fair; 0.41–0.60, moderate; 0.61–0.80, good; 0.81–1.00, very good.

### Comparison of the hookworm multiplex qPCR with the multiplex STH qPCR.

The hookworm multiplex qPCR showed very good agreement (Table 3) for the detection of both *N. americanus* and *Ancylostoma* spp. with the previously published multiplex STH qPCR.^11^ The multiplex STH qPCR failed to detect *N. americanus* infection in five samples, three of which constituted mixed infections with *A. ceylanicum* by the hookworm multiplex qPCR. The multiplex STH qPCR also failed to detect *A. ceylancium* in two samples, both classified as mixed infections with *N. americanus* by the multiplex hookworm qPCR. Among the five samples positive for *N. americanus* that were missed by the multiplex STH qPCR, two were also demonstrated positive by conventional PCR.

On the other hand, the hookworm multiplex qPCR failed to detect hookworm infection in two samples (one each for single *N. americanus* and *Ancylostoma* spp. infections) that were positive on the multiplex STH qPCR. The hookworm multiplex qPCR was superior to the multiplex STH qPCR for the detection of mixed infections of hookworm genera (Figure 2).

### Relationship between field-determined hookworm egg intensities (EPG) and multiplex hookworm qPCR.

Quantitative results derived from *N. americanus* fecal egg count and qPCR from field samples were compared to evaluate the relationship between infection intensity in EPG and Ct values (Figure 4). A moderate linear correlation between the average *N. americanus* egg counts measured by fecal flotation and Ct by qPCR was found (*R*^2^ = 0.6848). The conversion formula for Ct to egg intensities (EPG) was log_10_ EPG = −0.2161*x* + 6.61. The correlation between the average *A. ceylanicum* and *A. duodenale* fecal egg counts and average DNA concentration could not be determined because of the low number of egg-positive *A. ceylanicum* samples in this study.

On average, EPG estimates from Ct conversion formulas derived from the field-based fecal flotations were between 4 and 8.5 times less than those obtained for the experimentally seeded egg conversion values. The limitation of microscopy-based EPG estimates was particularly evident as field-derived *N. americanus* intensities reduced (Table 4).

**Table 4:** Comparing predicted intensities of *Necator americanus* (EPG) in field stool samples using Ct of field-based fecal flotation predicted formula log_10_ (EPG) = −0.2161*x* + 6.61, with Ct of seeded EPG conversion formula 4 × [log_10_ (EPG) = −0.1844*x* + 6.1145]

qPCR Ct value	EPG sodium nitrate flotation	Seeded formula predicted EPG	Flotation:PCR EPG ratio
15	2,344	8,924	1:3.8
16	1,419	5,837	1:4.1
17	864	3,817	1:4.4
18	525	2,497	1:4.8
19	319	1,633	1:5.1
20	194	1,068	1:5.5
21	118	698	1:5.9
22	72	457	1:6.4
23	44	299	1:6.8
24	27	195	1:7.4
25	16	128	1:7.9
26	10	84	1:8.5

Ct = cycle threshold; EPG = eggs per gram; qPCR = quantitative PCR. The seeded absolute egg count in 250 mg/μL DNA extract is multiplied by a factor of 4 for conversion to EPG.

## DISCUSSION

The newly developed multiplex hookworm qPCR assay was developed and validated against a well-described set of 192 field samples previously tested for hookworm infection using microscopy and both conventional and a multiplex STH qPCR. The hookworm multiplex qPCR was more sensitive than sodium nitrate flotation–based microscopy and conventional multiplex PCR for the detection of hookworms in stool. The assay showed very good agreement with the recently developed STH multiplex qPCR assay for the detection and genera-based differentiation of hookworms in human fecal samples. In addition, the multiplex hookworm qPCR developed in this study allowed for simultaneous detection and discrimination of all three hookworm species found in human stool, *N. americanus*, *A. ceylanicum*, and *A. duodenale*. The assay proved highly specific with no cross-reactivity observed for a range of 14 different intestinal parasites and with each other. The advantage of this qPCR over the other published diagnostic methods was its ability to detect mixed infections with different hookworm species in a field-based community survey at a higher sensitivity than conventional genus-based multiplex PCR, and a marginally higher sensitivity than the STH genus-based multiplex qPCR, making it ideal for monitoring hookworm infections in low-transmission settings. In total, two *A. ceylanicum* and three *N. americanus* infections were detected by the multiplex hookworm qPCR that could not be validated by either conventional or multiplex STH qPCR. Although there is a potential for false-positive results, the likelihood is low, given that each reaction was conducted in duplicate and individual assay efficiencies were consistently greater than 95%. This hookworm multiplex qPCR has the potential as a diagnostic tool to enable comprehensive investigation of the epidemiology of hookworm infection in humans in regions where *A. ceylanicum* and *A. duodenale* species may exist sympatrically.^16,19^ Distinction of these two *Ancylostoma* species in a community is becoming increasingly important. In the last decade, molecular-based diagnostic surveys in the Asia Pacific have increasingly identified *A. ceylanicum* as an emerging STH and zoonosis in communities where free-roaming dogs and cats act as important reservoirs for human infection.^4,20,21^ This qPCR allows for accurate, simultaneous, and high-throughput quantitative diagnosis of three hookworms, both zoonotic and anthroponotic in community surveys, thereby allowing targeted control programs to be implemented. This superior ability of multiplex PCR to detect coinfections is of key importance to disease burden, as individuals with multiple parasite infections are at an elevated risk of morbidity, especially since recent natural reports of confirmed patent human infections with *A. ceylanicum* produce chronic nausea, diarrhea, abdominal pain, melena, and hypereosinophilia.^22–24^ Despite the limitations associated with the low sensitivity of the Kato Katz, it remains the cornerstone of monitoring national STH control programs globally. As current global mass deworming campaigns aimed at interrupting STH transmission and eliminating morbidity expand, so will the reliance on more sensitive techniques such as qPCR. The economic limitations to qPCR are gradually being overcome by the ability to preserve and batch transport fecal samples at room temperature to central national laboratories with qPCR capabilities. This negates the need to dispatch teams of technicians and equipment to remote regions, thereby reducing costs associated with fieldwork. Further studies comparing costing and cost-effectiveness analyses of conventional microscopy and qPCR as a basis for monitoring STH control programs will prove valuable in future.

One of the primary goals of quantitative PCR is to link the qPCR data to the presently accepted indirect measurement of worm burden; that is, the infection intensity, measured by eggs per gram stool (EPG). Data comparing seeded hookworm eggs of known quantities and Ct values demonstrated excellent fit for *N. americanus*, *A. duodenale*, and *A. ceylanicum*. This provides the multiplex hookworm qPCR the ability to convert Ct values to EPG values for all three species of human hookworm by a simple formula that can be applied to epidemiological studies.

Quantitative PCR Ct values to field-based microscopy determined EPG values for *N. americanus* that also showed a moderate-to-good fit. Variability in the recovery of eggs from fecal samples subjected to fecal flotation and microscopic examination, as opposed to the accuracy of the qPCR assay, is the likely explanation of moderate to good, as opposed to excellent Ct to EPG correlations observed. On average, EPG estimates from Ct conversion formulas derived from the field-based fecal flotations were between 4 and 8.5 times less than those obtained for the experimentally seeded egg conversion values. The limitation of microscopy-based EPG estimates was particularly evident as flotation-derived *N. americanus* intensities reduced. The poor recovery of similar geohelminth parasites (ascarids, trichurids, and strongyles) in the feces of animals using sodium nitrate flotation has been proven in the past. For example, O’Grady and Slocombe^25^ found 50% of eggs trapped in the feces or retained in the strainer and 16–29% of eggs retained in the flotation solution.

Despite the ability of the multiplex hookworm qPCR to have excellent quantitative abilities, limiting factors for the application of absolute quantification of egg intensities on field-derived samples include confounding factors such as the degree of embryonation of hookworm eggs between time of defecation and time of stool refrigeration or fixation. In addition, DNA sourced from fragments of dead or dying worms in stool following natural death or anthelmintic treatment may also falsely elevate EPG estimates.

Obtaining sufficient quantities of purified eggs for each hookworm species (a limitation in this study) would allow a greater number of seeded aliquots of eggs to be enumerated by qPCR, thereby allowing the repeatability of the quantification experiment to be established and providing stronger confidence in the Ct to EPG conversion formulas. In this study, we were only able to subject two aliquots of eggs to DNA extraction for each hookworm species. Although the current multiplex qPCR is capable of detecting all three species of hookworm in stool, only two *A. duodenale*–positive DNA samples were available for inclusion in the present comparative study. Future application of this assay to regions endemic for *A. duodenale* will assist in confirming its ability to detect and quantify field-based microscopic egg counts for this hookworm species.

The amplification conditions for the multiplex hookworm qPCR were deliberately designed to coincide with the multiplex STH qPCR, capable of the additional detection and quantitation of *Ascaris* and *Trichuris* spp. in stool^11^ and with a previously published semiquantitative pentaplex qPCR for the detection of *G. duodenalis*, *Cryptosporidium* spp., *Entamoeba histolytica*, and *Strongyloides* spp. in stool.^11^ Future optimization and validation of these mixed target multiplex qPCR will allow for selection and simultaneous detection of a range of important STH helminth and enteric protozoa, which will facilitate tremendous flexibility for future parasite surveys.

In conclusion, this hookworm multiplex qPCR for the simultaneous detection of all three species of hookworm in human stool is more sensitive and better capable of detecting mixed hookworm species infection compared with conventional microscopy and PCR-based methods. The assay’s ability to be quantitative has the potential for use in monitoring the efficacy of hookworm control programs in low transmission settings and determining the efficacy of anthelmintics on individual species of hookworms, thereby directly allowing assessment of species-specific susceptibility to drugs and non-chemotherapeutic interventions.

## Supplementary Material

Supplemental figure
