# Supplementary material for: Development and Evaluation of a Multiplex Quantitative Real-Time Polymerase Chain Reaction for Hookworm Species in Human Stool
Source: Am J Trop Med Hyg. 2018 Sep 17;99(5):1186–93. doi: 10.4269/ajtmh.18-0276 (PMC6221243; doi:10.4269/ajtmh.18-0276)
Supplement: Supplementary file 1 [file tpmd180276.SD1.pdf]

```
A_ceylanicum_DQ780009       --------------CGTGCTAGTCTTCAGGACTTTGTCGGG-AAGGTTGG
A_caninum_JQ812694          TCCTGAGAAACCAACGTGCTAGTCTTCACGACTTTGTCGGGGAAGGTTGG
A_duodenale_EU344797        TCCTGAGAAACCAACGTGCTAGTCTTCACGACTTTGTCGGG-AAGGTTGG
                                          ************** ************ ********

A_ceylanicum_DQ780009       GAGTATCGCCCCCCGTTACAGCCCTACGTGAGGTGTCTATGTGCAGCAAG
A_caninum_JQ812694          GAGTATCGCCCACCGTTACAGCCCTATGTAAGGTGTCTATGTGCAGCAAG
A_duodenale_EU344797        GAGTATCGCCCCCCGTTATAGCCCTACGTAAGGTGTCTATGTGCAGCAAG
                            *********** ****** ******* ** ********************

A_ceylanicum_DQ780009       AGCCGTTCCTGGGTGGCGGCAGTGATTGCTGTGCGAAGTTCGCGTTTCGC
A_caninum_JQ812694          AGTCGTTACTGGGTGGCGGCAGTGATTGCTGTGCGAAGTTCGCGTTTCGC
A_duodenale_EU344797        AGTCGTTACTGGGTGACGGCAGTGATTGCTGTGCGAAGTTCGCGTTTCGC
                            ** **** ******* **********************************

A_ceylanicum_DQ780009       TGAGCTTTAGACTTGATGAGCATTGCATGAATGCCGCCTTACTGCTTGTG
A_caninum_JQ812694          TGAGCTTTAGACTTGATGAGCATTGCATGAATGCCGCCTTACTGCTTGTG
A_duodenale_EU344797        TGAGCTTTAGACTTGATGAGCATTGCATGAATGCCGCCTTACTGCTTGTG
                            **************************************************
```

Supplementary Figure 1. DNA sequence alignment of partial internal transcribed-1 spacer region of SSU rRNA gene of *Ancylostoma ceylanicum* (DQ780009), *Ancylostoma caninum* (JQ812694) and *Anyclostoma duodenale* (EU344797) displaying the location of the forward and reverse primers (bolded underline) and probes (double underlined) used in the hookworm real-time PCR.
